# The Toxicological Risk Assessment of Lead and Cadmium in *Valeriana officinalis* L., radix (Valerian root) as Herbal Medicinal Product for the Relief of Mild Nervous Tension and Sleep Disorders Available in Polish Pharmacies

**DOI:** 10.1007/s12011-021-02691-5

**Published:** 2021-04-01

**Authors:** Kamil Jurowski, Maria Fołta, Barbara Tatar, Mehmet Berkoz, Mirosław Krośniak

**Affiliations:** 1 Institute of Medical Studies, Medical College, Rzeszów University, Al. mjr. W. Kopisto 2a, 35-959 Rzeszów, Poland; 2 Department of Food Chemistry and Nutrition, Medical College, Jagiellonian University, Medyczna 9, 30-688 Kraków, Poland; 3 Department of Biochemistry, Faculty of Pharmacy, Van Yuzuncu Yil University, 65080 Van, Turkey

**Keywords:** Valerian root, Elemental impurities, ICH Q3D, PDE, Toxicological analysis

## Abstract

Studies related to the toxicological risk assessment (TRA) of heavy metal impurities (HMIs) in pharmaceuticals are an important issue but there is a lack of refereed literature around the safety of *Valeriana officinalis* L., radix (Valerian root) as herbal medicinal product (HMP) for the relief of mild nervous tension and sleep disorders according to lead and cadmium impurities. The aim of the study was to estimate the TRA of lead and cadmium in *Valeriana officinalis* L., radix (Valerian root) as HMP (*n* = 5) available in Polish pharmacies. In the case of herbal pharmaceuticals, it is particularly important to control the level of HMIs accumulated during the plant’s growth. Perhaps, the exposure for a single dose is not relevant; however, justification of our studies is a fact that herbal therapies are usually long term. Therefore, even small HMI doses as present in particular plant may accumulate in patient body over a long period of time. Levels of lead and cadmium were measured by electrothermal atomization atomic absorption spectrometry. The levels of lead and cadmium as HMIs (independently of the producer and declared composition) are quite similar. Our results are satisfactory, confirming the safety of *Valeriana officinalis* L., radix (Valerian root) as herbal medicinal product for the relief of mild nervous tension and sleep disorders available in Polish pharmacies according to ICH guideline Q3D. To the best of our knowledge, this paper is the first study about lead and cadmium content as HMIs in HMP containing *Valeriana officinalis* L., radix (Valerian root).

**Supplementary Information:**

The online version contains supplementary material available at 10.1007/s12011-021-02691-5.

## Introduction

**Fig. 1:**
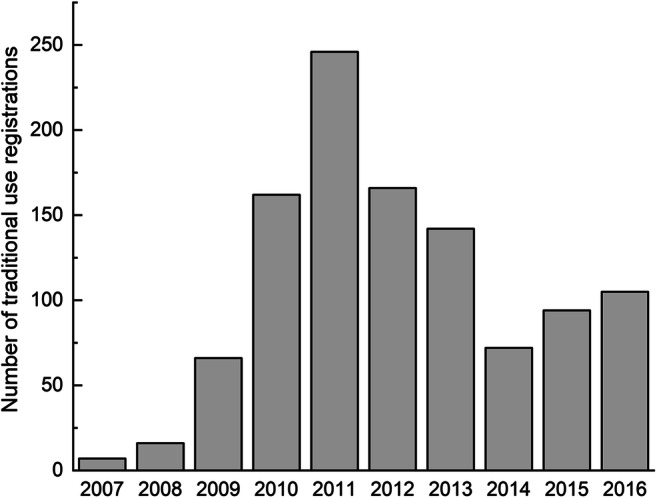
Number of traditional use registrations for monocomponent HMP in the EU grouped by year of registration (based on [2])

Herbal medicinal product (HMP) can be defined as “any medicinal product, exclusively containing as active ingredients one or more herbal substances or one or more herbal preparations, or one or more such herbal substances in combination with one or more such herbal preparations” [1]. Based on number of traditional use registrations per year for HMP in the EU, it can be stated that monocomponent HMP are still popular for use (Fig. 1 based on [2]).

From this point of view, very intriguing plant applied in pharmaceutical industry as monocomponent HMP is *Valeriana officinalis* L. (valerian root). Valerian root is a perennial herbaceous plant (*Valerianaceae* family) that has been widely used in therapy since ancient times. In many countries (especially across Europe), Valerian root is a popular HMP for the relief of mild nervous tension and sleep disorders [3]. However, the evidence that valerian root extracts are sedative in people has been equivocal for many decades. This HMP have been shown in human to improve sleep quality [4], to reduce latency to fall asleep [5], and to diminish feelings of somatic arousal during a social stress situation [6, 7]. Additionally, *V. officinalis* L. extract was found to contain material binding to the central benzodiazepine receptor although being without other benzodiazepine-like properties [8].

From toxicological point of view, very important problem is control of heavy metals impurities (HMIs) in pharmaceutical herbal products (especially, lead and cadmium). Due to the varied bioavailability of HMIs in soil, their level in raw materials (herbs) may vary significantly [9, 10]. The control of HMIs in HMP is relevant problem and should meet the standards of directive International Conference on Harmonisation’s Q3D Guideline on Elemental Impurities [11]. However, there is very little information about toxicological risk assessment (TRA) of HMP sold in the UE market (especially in pharmacies). Hence, scientific articles about HMIs in HMP are extremely desirable but surprisingly are not published often.

Therefore, the aim of this article was TRA of lead and cadmium in *V. officinalis* L., radix (Valerian root) as HMP for the relief of mild nervous tension and sleep disorders available in Polish pharmacies. Our TRA of lead and cadmium included five HMP with *V. officinalis* L., radix (Valerian root) available in Polish pharmacies. The choice of metals was justified by their potential possibility of occurrence in this herb (based on review of the literature [10]) and our apparatus capabilities.

## Materials and Methods

### Samples and Chemicals

The five *V. officinalis* L., radix (Valerian root) as HMP were analyzed for the relief of mild nervous tension and sleep disorders. The choice of HMP was justified by:

1. availability in Polish pharmacies as representative example of European country due to this herb (Valerian root is one of the major herbs processed by the pharmaceutical industry, with a total annual harvest exceeding 1000 tones in Poland [10]);
2. the fact that Valerian root is an example of monocomponent HMP (the exclusion of other sources of metallic impurities);
3. literature overview about HMIs in Valerian root [10].

To maintain the highest methodological standards, each HMP was coded (A, B, and so on). A short description of the samples is given in the Supplementary Materials 1. To minimize any potential impurities from other sources, all steps during the sampling procedure were carried out in plastic equipment. Additional treatment of samples (like homogenization and digestion) was not necessary, because all HMP were liquid samples (valerian drops). Hence, in situ analysis was applied at measurement step (see sample analysis section).

The water for the experimental work was demineralized water. Nitric acid (65%) was of spectroscopic grade (Merck SupraPur, Darmstadt, Germany). Standard solutions of lead (Pb standard solution traceable to SRM from NIST–Pb(NO_3_)_2_ in 0.5 mol·L^−1^ HNO_3_, 1000 mg L^−1^ Cd CertiPUR®, catalog product: 19776.0500) and cadmium (Cd standard solution traceable to SRM from NIST–Cd(NO_3_)_2_ in 0.5 mol·L^−1^ HNO_3_, 1000 mg L^−1^ Cd CertiPUR®, catalog product: 1.19777.0500) were prepared by dilution of certified standard solutions, 1000 μg·L^−1^ MERC of corresponding metal ions.

### Instrumentations

A Perkin-Elmer 5100 ZL atomic absorption spectrometer (Perkin-Elmer, Norwalk, CT, USA) with Zeeman background correction and with electrothermal atomization (ETAAS technique) was used for determination of lead and cadmium using the appropriate hollow cathode lamps. All detailed information about instrumentation is described in Supplementary Materials 2.

### Toxicological Risk Assessment Approach

**Fig. 2:**
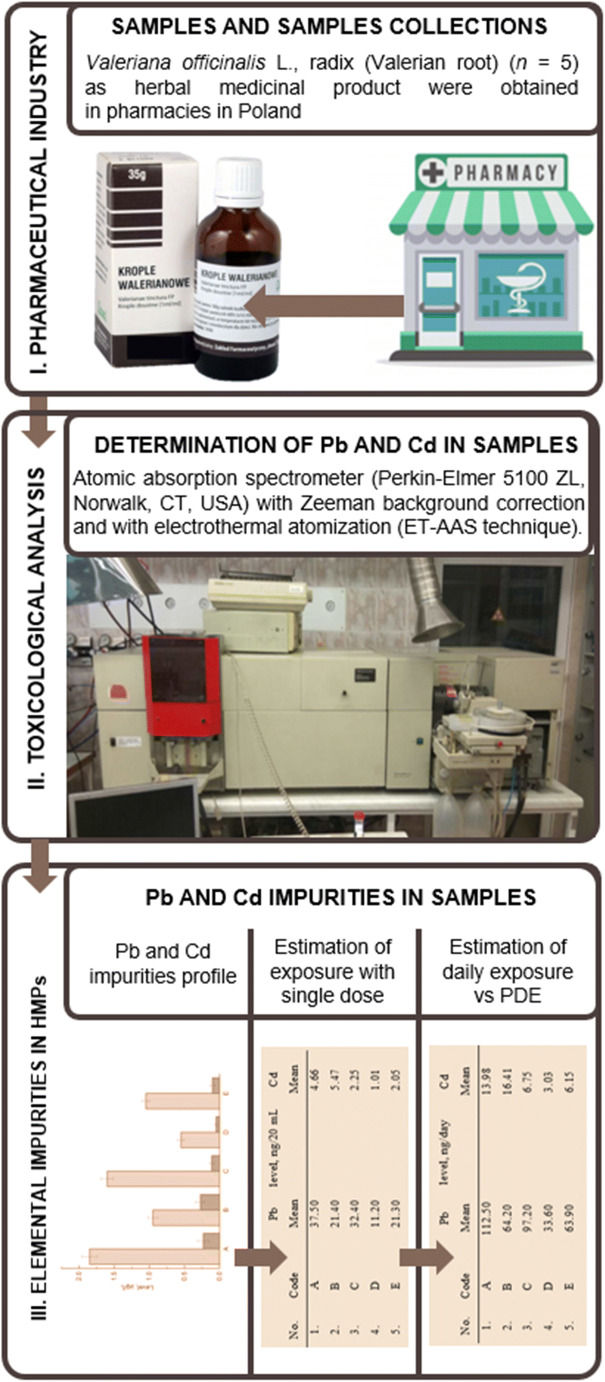
The basic workflow of the applied toxicological risk assessment of lead and cadmium in *V. officinalis* L., radix (Valerian root) as HMP available in Polish pharmacies.

The TRA of metal contamination is always based on toxicological analysis of each element in analyzed samples. Applied TRA of lead and cadmium procedure was modification (in situ analysis - omission of the samples preparation step) of our methodology published earlier [12, 13]. All steps of the measurement are summarized in the graphical workflow of Fig. 2.

Pyrolytically coated graphite tubes with L’vovs platforms were applied. All detailed information about analytical measurement, calibration strategy, and quality control are included in Supplementary Materials 2.

### Statistical Analysis

Data were analyzed using Educational Analysis Set SAS® 9 and Origin 2021 Pro licensed by the Jagiellonian University in Krakow. The resultant data of five independent replicates were expressed as the mean ± standard error.

## Results and Discussion

### The Heavy Metal Impurity Profile of Herbal Medical Products with *V. officinalis* L., radix (Valerian Root)

**Fig. 3:**
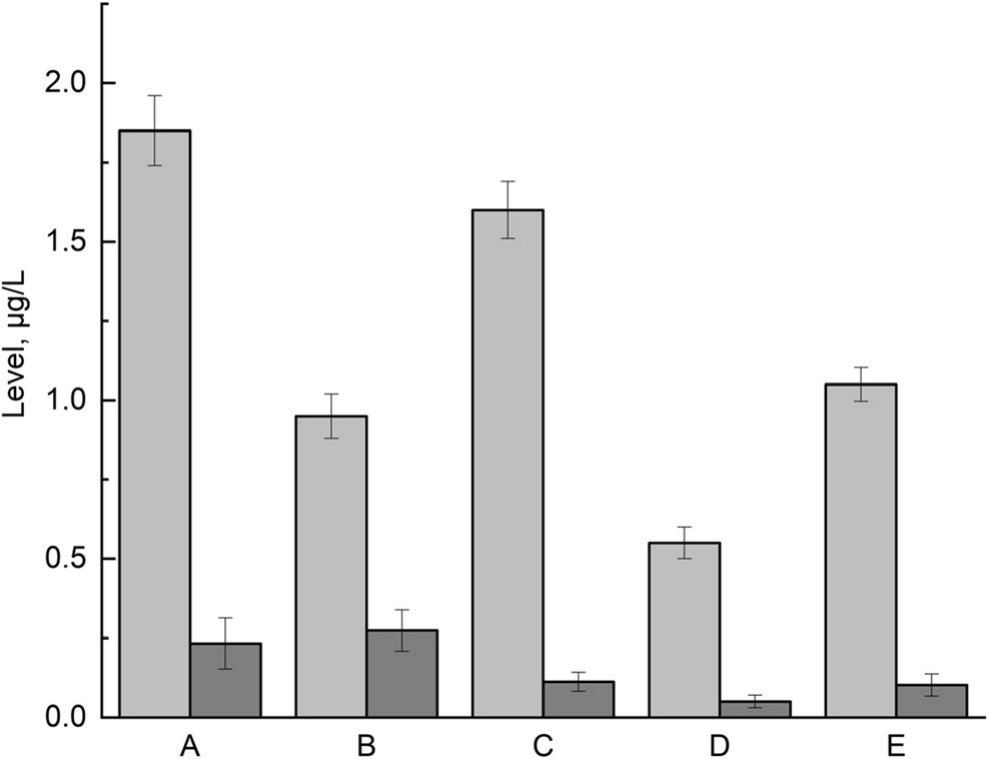
The heavy metal impurities’ profile of investigated herbal medical products samples (A, B, C, D, and E)

The levels of lead and cadmium in all HMP samples (*n* = 5; A–E) are shown in Fig. 3 as the HMI profile of a HMP sample, level per L of sample. Generally, lead and cadmium were present in all analyzed HMP on very low level; lead levels were approximately eight times higher (mean = 1.20 μg/L) than cadmium levels (mean = 0.15 μg/L).

The highest level of lead was in sample A (1.85 ± 0.15 μg/L) and the lowest level was in sample D (0.55 ± 0.08 μg/L). All samples contained levels of cadmium below the permissible limit set by FAO/WHO for medicinal herbs and plants in different countries (WHO recommendation, 10 mg/kg) [14]. Additionally, considering the concentration limits for lead as HMIs in pharmaceuticals via oral route recommended by directive ICH Q3D (0.5 μg/g [11]), all of the samples analyzed meet the guidelines.

On the other hand, the cadmium levels were similar for all HMP (0.103–0.275 μg/L), except sample D (0.051 μg/L, two times lower than lowest cadmium level in other samples). All samples contained levels of cadmium below the permissible limit set by FAO/WHO for medicinal herbs and plants in different countries (WHO recommendation, 0.3 mg/kg) [14]. Based on permitted concentrations for cadmium as HMIs in pharmaceutical products (oral concentration) recommended by directive ICH Q3D (0.5 μg/g [11]), all of the HMP meet the guidelines.

### Estimation of HMI Exposure for One-Time Administration (Single Dose) of HMP with *V. officinalis* L., radix (Valerian Root)

Level of lead and cadmium impurities including one-time administration (single dose) of HMP are needed for the assessment of these metals’ exposure in daily intake of applied pharmaceuticals. Based on posology and method of administration described in monograph on *V. officinalis* L., radix by European Medicine Agency (EMA/HMPC/150848/2015) [4], there are many ways to use these products. Based on traditional use described in mentioned monograph, to aid sleep, a single dose (20 mL) should be administered half to 1h before bedtime with an earlier dose during the evening if necessary. Hence, based on earlier results (Fig. 3.), we calculated levels of lead and cadmium in 20 mL of each HMP. The obtained results of lead and cadmium levels considering the one-time administration of HMP are shown in Table 1.

**Table 1:** The levels of lead and cadmium to which the patient is exposed for one-time administration of the herbal medical product with *V. officinalis* L., radix (Valerian root) (ng/20 mL)

Sample		Level, ng/20 mL	
		Pb	Cd
No.	Code	Mean	Mean
1.	A	37.50	4.66
2.	B	21.40	5.47
3.	C	32.40	2.25
4.	D	11.20	1.01
5.	E	21.30	2.05

### Daily Exposure of Lead and Cadmium in Herbal Medical Products with Valerian Root

Based on information in the monograph on *V. officinalis* L., radix by European Medicine Agency (EMA/HMPC/150848/2015) [4], the frequency of application should be no more than three times per day. The daily exposure of Pb and Cd from all investigated HMP is shown in Table 2.

**Table 2:** The estimated daily exposure of lead and cadmium to which the patient is exposed for daily administration of the herbal medical product with *V. officinalis* L., radix (Valerian root) (ng/day)

Sample		Level, ng/day	
		Pb	Cd
No.	Code	Mean	Mean
1.	A	112.50	13.98
2.	B	64.20	16.41
3.	C	97.20	6.75
4.	D	33.60	3.03
5.	E	63.90	6.15

The estimated daily exposure to lead is at a similar level (33.60–112.50 ng/day). Considering the Integrated Exposure Uptake Biokinetic (IEUBK) Model [15], based on the assumption of 100% absorption (Valerian root is an example of monocomponent HMP – no other sources of metallic impurities), the oral intake of lead is 5.0 μg/day. Hence, PDE (permitted daily exposure) for this heavy metal should be used as 5.0 μg/day [11]. The results of our TRA approach show that all analyzed HMP are characterized by results below PDE value (highest result 0.1125 ug/day).

On the other hand, the estimated daily exposures of cadmium are at a very variable level (3.03–16.41 ng/day). The key point for TRA of cadmium by oral route is renal toxicity [16]. Because many studies about oral exposure to cadmium in rats and mice showed no evidence of carcinogenicity, the renal toxicity endpoint was used by EMA to establish the oral PDE value as 5.0 μg/day [11]. Our results indicate that all analyzed HMP are characterized by results extremely lower than PDE value for cadmium (about 360–1600 times lower).

## Conclusions and Recommendations

The level of investigated HMIs in all of the HMP with *V. officinalis* L., radix (Valerian root) occurs at a very low level: levels of Pb range from 0.55 to 1.85 μg/L; levels of Cd range from 0.051 to 0.27 μg/L. Their content in a single administration (single dose) is also very low (ng/20mL) and is not a threat to patients. The estimated daily exposure (ng/day) of lead and cadmium meets the standards of directive ICH Q3D according to HMIs (all results were below established oral PDE for each heavy metal, i.e., < 5.0 μg/day). It can be concluded that our results show that all analyzed HMP with Valerian root available in pharmacies in Poland should not represent any health hazard to the patients due to lead and cadmium levels.

Since our TRA of daily exposure of lead and cadmium as HMIs provide pioneer data, they may be valuable for other investigators and manufacturers. Due to the fact that these kinds of studies are very rare, it would be valuable to carry out a broader study considering other HMP (for example from other countries) to build upon this data like studies recently described by Jurowski et al. [17, 18].

## Supplementary Information

ESM 1 (DOCX 15 kb)

ESM 2 (DOCX 18 kb)

## Abbreviations

AAS: Atomic absorption spectrometry technique
EI: Elemental impurities
ET AAS: Electrothermal atomization atomic absorption spectrometry
HMP: Herbal medicinal product
HMIs: Heavy metal impurities
ICH Q3D: International Council for Harmonization of Technical Requirements for Pharmaceuticals for Human Use
PDE: Permitted daily exposure
TRA: Toxicological risk assessment
SD: Standard deviation

## Declarations

This article does not contain any studies with human participants or animals performed by any of the authors.
