# Supplementary material for: The Toxicological Risk Assessment of Lead and Cadmium in Valeriana officinalis L., radix (Valerian root) as Herbal Medicinal Product for the Relief of Mild Nervous Tension and Sleep Disorders Available in Polish Pharmacies
Source: Biol Trace Elem Res. 2021 Apr 1;200(2):904–9. doi: 10.1007/s12011-021-02691-5 (PMC8738358; doi:10.1007/s12011-021-02691-5)
Supplement: Supplementary file 1 — (DOCX 15 kb) [file 12011_2021_2691_MOESM1_ESM.docx]

**Supplementary materials 1.** Short description of analyzed samples

| Sample | | description from European Pharmacopoeia | license | note |
| --- | --- | --- | --- | --- |
| No. | Code |  |  |  |
| 1. | A | *Valerianae tinctura* | IL-0969/LN | OTC |
| 2. | B | *Valerianae tinctura* | 14040 | OTC |
| 3. | C | *Valerianae tinctura* | IL-0025/LN | OTC |
| 4. | D | *Valerianae tinctura* | R/6696 | OTC |
| 5. | E | *Valerianae tinctura* | IL-2663/LN | OTC |

OTC - over-the-counter
