# Supplementary material for: The Toxicological Risk Assessment of Lead and Cadmium in Valeriana officinalis L., radix (Valerian root) as Herbal Medicinal Product for the Relief of Mild Nervous Tension and Sleep Disorders Available in Polish Pharmacies
Source: Biol Trace Elem Res. 2021 Apr 1;200(2):904–9. doi: 10.1007/s12011-021-02691-5 (PMC8738358; doi:10.1007/s12011-021-02691-5)
Supplement: Supplementary file 2 — (DOCX 18 kb) [file 12011_2021_2691_MOESM2_ESM.docx]

I. Analytical procedure and parameters

The linear range of calibration function reached from the detection limit up 0.0; 1.0; 2.0; 5.0; 10.0 Pb µg/L and 0.0; 0.5; 1.0; 2.0 µg/L for Cd. The values of the correlation coefficient (*R*) are good indicators of the linearity for AAS instruments giving precise and accurate results [[7](#_ENREF_7), [8](#_ENREF_8)]. The correlation coefficients obtained (0.998 for Pb and 0.998 for Cd) indicated that the analyses were both precise and accurate.

The recoveries were 98.2 % for Pb and 97.5 % for Cd. The recoveries were calculated as the quotient of the determined level and the known amount of the determined element expressed as a percentage. The calculated limits of detection (LODs) were 0.45 µg/L for Pb and 0.15 µg/L for Cd. The calculated limits of quantification (LOQs) were 0.95 µg/L for Pb and 0.34 µg/L for Cd.

II. Conditions of apparatus

1. The AAS operating conditions.

| Operating parameters | heavy metal | |
| --- | --- | --- |
|  | Pb | Cd |
| Wavelength [nm] | 283.3 | 228.8 |
| Slit width [nm] | 0.7 | 0.7 |
| Lamp current [mA] | 8 | 5 |
| Optimum working range [µg/kg] | 1.0-10.0 | 0.02-0.20 |

2. Time-temperature program in the graphite furnace atomic absorption spectrometer in Pb and Cd determination.

Pb

| Step | Temperature [°C] | Ramp [s] | Hold [s] | Gas Flow [mL/min] |
| --- | --- | --- | --- | --- |
| 1 | 120 | 1 | 30 | 250 |
| 2 | 950 | 10 | 20 | 250 |
| 3 | 1450 | 0 | 5 | 0 |
| 5 | 2400 | 1 | 2 | 250 |

Cd

| Step | Temperature [°C] | Ramp [s] | Hold [s] | Gas Flow [mL·min^-1^] |
| --- | --- | --- | --- | --- |
| 1 | 120 | 10 | 25 | 250 |
| 2 | 300 | 5 | 15 | 250 |
| 3 | 1600 | 0 | 3 | 0 |
| 5 | 2400 | 1 | 2 | 250 |
